# The Hemisphere of the Brain in Which a Stroke Has Occurred Visible in the Heart Rate Variability

**DOI:** 10.3390/life12101659

**Published:** 2022-10-20

**Authors:** Joanna Aftyka, Jacek Staszewski, Aleksander Dębiec, Aleksandra Pogoda-Wesołowska, Agata Kowalska, Anna Jankowska, Jan Żebrowski

**Affiliations:** 1 Faculty of Physics, Warsaw University of Technology, Koszykowa 75, 00-662 Warsaw, Poland; 2 Clinic of Neurology, Military Institute of Medicine, Szaserow 128, 04-141 Warsaw, Poland

**Keywords:** heart rate variability, ischemic stroke, sample entropy, brain hemisphere, complexity, nonlinear analysis

## Abstract

The aim of this study was to assess whether heart rate variability (HRV) could predict which hemisphere of the brain was affected during an acute ischemic stroke (AIS). To achieve this goal, we compared HRV between patients with a right (RH) and left hemispheric (LH) stroke. The studied group consisted of 64 patients with AIS (25 with RH and 39 with LH stroke, with a mean age of 64 ± 12 and 66 ± 13, *p* = 0.3, respectively) using 24 h Holter ECG records at *NN* intervals performed at a mean of 4.3 ± 2 days following their AIS. Standard linear methods were used to analyze HRV in the time and frequency domains, as well as nonlinear methods, including sample entropy, detrended fluctuation analysis, and asymmetry measures. Patients with an LH stroke had significantly greater values for sample entropy compared to subjects with an RH stroke (1.31 ± 0.53 vs. 0.92 ± 0.46, *p* = 0.003, Bonferroni-corrected *p* = 0.033, effect size = 0.8). The LH stroke group also had higher RMSSD (113 ± 81 vs. 76 ± 61, *p* = 0.06), pNN50 (33.35 ± 28.54 vs. 18.52 ± 23.75, *p* = 0.02), and HF_nu_ (48.42 ± 16.41 vs. 42.66 ± 17.88, *p* = 0.11) values, when compared to the RH group, which was possibly related to higher activity in the parasympathetic system in the LH group. Conversely, subjects with RH stroke had higher LF_nu_ (57.34 ± 17.88 vs. 51.58 ± 16.41, *p*-value = 0.11) and LF/HF ratios (2.24 ± 2.87 vs. 1.68 ± 2.50, *p*-value = 0.11), which were likely related to higher activity in the sympathetic nervous system, when compared to the LH stroke group. Our pilot study demonstrated that patients with RH stroke had lower HRV complexity than those with LH stroke, indicating that HRV could be useful in the discrimination of hemispheric involvement in AIS.

## 1. Introduction

Stroke is the second leading cause of mortality worldwide, with a high estimated lifetime risk between 8% and 10% [1]. The pathophysiology of ischemic stroke is complex and related to numerous processes, such as energy failure, loss of cellular ion homeostasis, increased intracellular calcium levels, excitotoxicity and cytotoxicity, disruption of the blood–brain barrier, and activation of glial cells [2]. These events are interrelated and can all lead to ischemic necrosis, which occurs in the severely affected ischemic core regions. However, the human body is not a collection of separate systems but an integrated network of many physiological systems that constantly interact with each other. According to the concept of the physiological network, research is currently being carried out in which the mutual interactions of two or more systems in the human body are observed [3]. In humans, a stroke in either hemisphere of the brain has been shown to result in changes to autonomic mechanisms. However, the localization of a stroke may have differential effects. Right-hemisphere lesions are linked to a greater occurrence of supraventricular tachycardia, a decrease in cardiac parasympathetic activity, and a rise in sympathetic tone. Moreover, experimental and human research has revealed that the insular cortex, anterior cingulate gyrus, hypothalamus, and amygdala are involved in regulating the central autonomic nervous system (ANS). Therefore, acute or chronic ischemia involving these regions may result in significant ANS dysfunction [4,5]. Importantly, ANS and cardiac dysfunction may occur following acute vascular brain injury without any evidence of primary heart disease [6].

HRV analysis is a simple noninvasive bedside method to assess sympatho-vagal balance and has been used extensively to assess autonomic dysfunction following myocardial infarction and acute or chronic stroke [7]. Many studies have shown that patients with reduced or abnormal HRV have an increased risk of stroke [8,9], poststroke mortality [10,11], disability, and other medical complications [12,13]. However, due to the complex nature of the brain–heart axis and the different methods used for analyzing HRV dynamics (time-domain, spectral, and nonlinear measures), the clinical and pathophysiological importance of these findings has not been clearly established [14]. The classic, commonly used methods for analyzing HRV are linear measures in the time and frequency domains [15]. However, it is postulated that HRV is better characterized by cardiovascular regulation mechanisms, which interact nonlinearly [16]. Nonlinear methods are more reliable as indicators for the performance of physiological systems [17]. Therefore, the optimal characterization of normal and abnormal HRV could likely be based on a combination of these different metrics, rather than a single metric. The comparison of HRV in left- or right-sided hemispheric injury has attracted much attention, and some evidence exists regarding the role of forebrain lateralization in cardiovascular autonomic regulation among patients with cerebrovascular disease [18]. Colivicchi et al. [19] revealed that patients with right-sided insular damage, compared to other stroke patients, had significantly lower values for the standard deviation of all normal-to-normal intervals (SDNN), lower root mean square values for their differences (RMSSD), and a higher low-frequency to high-frequency ratio (LF/HF) (*p* < 0.05). Naver et al. [20] found that right-sided stroke subjects had reduced respiratory HRV compared to left-hemisphere patients and the control group. Constantinescu et al. [21] demonstrated that patients with a left-hemisphere stroke presented higher RMSSD, pNN50, and normalized HF values than those with a right-hemisphere injury. Another study of patients who experienced their first-ever ischemic stroke and had no pre-existing cardiac disease observed a more pronounced decrease in HRV measurements in those with right-sided insular involvement [22]. Conversely, Laowattana et al. showed increased rates of cardiac events in patients with left insular infarction compared to other strokes [23]. Additionally, other researchers found no differences when using HRV linear analysis in the time domain (RMSSD, pNN50, and SDNN) and frequency domain (LF, HF, VLF, and LF/HF) between right- and left-hemisphere stroke [24]. Thus, the relationship between the frequency of cardiac autonomic derangement and the hemisphere of stroke injury still remains controversial.

In addition to its practical importance, knowledge regarding the effects of hemispheric brain damage would make it easier to understand the functions of the brain–heart axis and the influence of stroke lateralization on cardiovascular outcomes. Depending on the time of the examination, in about 20–30% of patients with acute ischemic stroke (AIS), there were no early abnormalities observed in the computed tomography (CT) scans of the brain within the first 24 h following stroke onset. In some patients, there were also no pronounced focal changes in their physical examination [25]. The presence of an ischemic focus can be easily confirmed via a magnetic resonance imaging (MRI) examination. However, the availability of this test is limited in an acute setting, and the time needed to perform the test can extend up to several days. In addition, not all patients may be able to undergo an MRI examination (e.g., due to stroke-related psychomotor agitation, the presence of metal parts in the body, or claustrophobia). Therefore, the easier and more available HRV analysis—especially single or serial ECG analysis in the acute or hyperacute phase of the stroke, most notably during the prehospital or early in-hospital stage with no neuroimaging facilities—could become a useful tool for the assessment of acute stroke.

In our study, we aimed to determine whether it was possible to recognize the laterality of a stroke (right hemisphere [RH] or left hemisphere [LH]) using HRV analysis as the only discrimination factor. We evaluated if there were differences in the linear and nonlinear parameters of HRV between patients with RH stroke and LH stroke. The results of our study may help define the role of HRV analysis in predicting the location of a stroke.

## 2. Materials and Methods

### 2.1. Participants

This was a retrospective study, and the data contained in the article were obtained from routine diagnostic tests conducted among patients admitted to the Stroke Unit. Because the purpose of our study was to identify differences in the HRV records between right- and left-hemisphere stroke patients, our study did not include a healthy control group. The study group consisted of 64 consecutive patients with confirmed AIS who were hospitalized at the Comprehensive Stroke Center of the Military Institute of Medicine in Warsaw between 2010 and 2019, fulfilling the pre-established eligibility criteria. The inclusion criteria consisted of (1) a 24 h Holter ECG examination performed in the acute phase (<7 days after onset) of ischemic stroke; (2) a neuroimaging examination (CT on days 0 and 1, and/or MRI on day 0) to confirm AIS lesions, determine the stroke’s hemispheric localization, and assess the volume of AIS using the Alberta stroke program early CT score (ASPECTS) [26]; and (3) anterior large vessel occlusion stroke. The exclusion criteria for patients were hemorrhagic or transient ischemic stroke (TIA) or the presence of any ANS disorders (e.g., atypical parkinsonism). Among all the patients, control neuroimaging was performed in the case of neurologic deterioration to rule out secondary intracranial hemorrhaging (sICH). An ASPECTS score less than or equal to 7 predicted a more severe stroke with worse functional outcomes at 3 months, as well as sICH. All the subjects received standard stroke diagnosis, treatment, and rehabilitation according to the guidelines [27]. A total of 67 patients were screened, and 3 subjects with brainstem or cerebellar stroke were excluded from the study. Ultimately, 64 patients qualified for the analyses, including 25 patients with diagnosed RH stroke and 39 patients with LH stroke.

We carried out this study in accordance with the Declaration of Helsinki. The electronic database was decoded, and the patient identification data were scrambled to ensure confidentiality; informed consent was thus exempted. The evaluation of all studies, including Holter ECG monitoring or HRV interpretation, was blinded from the clinical data form the CT or MRI scans. This study was evaluated and approved as an internal study at the Military Institute of Medicine in Warsaw (no.: 00574) and by the Institutional Review Board of Military Institute of Medicine in Warsaw (no.: 20WIM/2020 at 22 April 2020).

### 2.2. Heart Rate Variability

The 24-h HRV recordings were evaluated using a Reynolds Medical Holter ECG Lifecard CF device. All examinations were inpatient recordings and performed at a mean of 4.3 ± 2 days following stroke onset (LH 4.4 ± 2 vs. RH 4.3 ± 1.5 days; *p* = 0.61). Subjects with LH and RH stroke had a similar baseline functional disability and day–night activity patterns related to the organization of hospital care (night hours from 10:00 p.m. to 6:00 a.m. and the day hours from 6:00 a.m. to 10:00 p.m.). The sampling frequency was 128 Hz. All recordings were automatically analyzed and then visually scanned to determine if the records were appropriately classified. All misclassified beats or other artifacts were manually corrected by an experienced physician, and the recordings were then exported into text files containing the duration of each cardiac cycle and annotation of the beat type (sinus, supraventricular, ventricular, or technical artifacts). Signal samples with non-normal beats were removed from the signal before starting the analyses. A further fragment of the heart rhythm (marked as normal beats after ectopy/artifacts) was attached to the fragment preceding the ectopy/artifacts. As a result, a signal was created that contained only normal stimuli. Signal samples with non-normal beats were removed from the signal before starting the analyses. A further fragment of the heart rhythm (marked as normal beats after ectopy/artifacts) was attached to the fragment preceding the ectopy/artifacts. As a result, a signal was created that contained only normal stimuli. The normal-to-normal intervals were analyzed in the HRV analysis. Both methods of linear analysis in the time and frequency domains, as well as nonlinear analysis, were used in this work.

### 2.3. Linear Methods

#### 2.3.1. Time Domain

There are many methods for linear HRV analysis in the time domain. For this study, we selected commonly used measures, such as meanNN, SDNN, RMSSD, and pNN50 [15]. The first measure is the mean duration of the *NN* interval (meanNN) and is expressed in milliseconds. The average value of *NN* intervals was determined for the entire record as the sum of all successive *NN* intervals divided by the total number of *NN* intervals in the record. SDNN refers to the deviation in the *NN* intervals relative to the meanNN and is expressed in milliseconds. This measure shows how much the points are scattered around the mean of the signal. The RMSSD measure is the root mean square-successive differences between successive *NN* intervals. In the first step of the analysis, we calculated the difference in time between successive heartbeats in ms. Then, each value was squared, and the result was averaged. In the last step, we evaluated the square root of the result. Here, the pNN50 indicates the percentage share of such samples throughout the entire recording, where the difference between the adjacent *NN* intervals was greater than 50 ms.

The parameters of linear analysis in the time domain were determined in the Python 3.8 environment using the hrvanalysis.get_time_domain_features package.

#### 2.3.2. Frequency Domain

Frequency domain linear analysis was expressed as HF_nu_ and LF_nu_ measures [15]. These measures reflect the power spectra in the high-frequency range (0.15–0.4 Hz) and low-frequency range (0.04–0.15 Hz), respectively. Power spectra are given in normalized units, where the numerator is the corresponding power spectrum HF or LF, and the denominator is the sum of both power spectra (LF + HF). Values are expressed as percentages. Additionally, we determined the ratio of the power spectrum in the low-frequency range to the power spectrum in the high-frequency range (LF/HF).

The parameters of linear analysis in the time domain were determined in the Python 3.8 environment using the hrvanalysis.get_frequency_domain_features package.

### 2.4. Nonlinear Methods

Two measures of heart rhythm asymmetry were calculated in the present study: the Guzik index (GI) [28] and the Porta index (PI) [29]. Using this method, the lines of identity must first be determined on the Poincaré plot, i.e., the line on which the points *NN_i_* = *NN_i_*_+1_ lie. The Guzik index specifies the distance from the points above the identity line to the identity line, divided by the distance of all points in the Poincaré plot to the identity line, except for the points on the identity line (Equation (1)):

(1) $$GI=\frac{\sum_{i=1}^{C\left(P_{i}^{+}\right)}{\left(D_{i}^{+}\right)}^{2}}{\sum_{i=1}^{N-1}{\left(D_{i}\right)}^{2}}*100\%$$

where the distance is determined by Equation (2):

(2) $$D_{i}=\frac{\left|NN_{i}-NN_{i+1}\right|}{\sqrt{2}}$$

The Porta index, unlike the GI, does not consider points on the line of identity. The Porta index describes the ratio of the number of points on the Poincaré chart above the identity line to the sum of the points above and below the identity line (Equation (3)):

(3) $$D_{i}=\frac{\left|NN_{i}-NN_{i+1}\right|}{\sqrt{2}}$$

Both methods (GI and PI) are based on standard Poincaré plots (lag = 1), i.e., graphs of the relationship between the *NN* intervals and intervals of 1 shifted *NN +* 1.

For the purpose of the present study, we also evaluated the results for sample entropy [30]. Sample entropy is a measure of complexity expressed as the natural logarithm of the number of matches with length *m +* 1 with the *i*th template (*A_i_*) to the number of matches with length *m* with the *i*th template (*B_i_*) (Equation (4)):

(4) $$Sample\;Entropy\left(m,r\right)=-ln\left(\frac{\sum^{\;}A_{i}}{\sum^{\;}B_{i}}\right)=-ln\left(\frac{\sum^{\;}A}{\sum^{\;}B}\right)$$

The values of the parameter ‘*m*’, i.e., the length of the pattern, and ‘*r*’, i.e., the tolerance parameter, were assumed, respectively, as *m* = 2 and *r* = 0.2 * std [30].

Another two measures of nonlinear HRV analysis come from detrended fluctuation analysis (DFA) [31]. In this method, the two scaling factors of α_1_ and α_2_ are determined. The first exponent contains information on short-range correlations (4–16 consecutive *NN* intervals). The exponent α_2_ evaluates the long-range sequences (17–64 *NN* intervals).

Nonlinear HRV analysis was performed in Python 3.8 using the pyentrp.entropy packages for sample entropy and pyhrv.nonlinear to determine the two scaling factors, α_1_ and α_2_, from the detrended fluctuation analysis. The Guzik and Porta indexes were determined based on self-written code in accordance with the mathematical formulas from [28,29].

### 2.5. Statistical Analysis

For each of the analyzed HRV variables, a Shapiro–Wilk test was performed to verify the normality of the variable distributions. All measures did not have normal distributions, so a nonparametric Mann–Whitney test was used for statistical analysis; the results with *p* < 0.05 were considered statistically significant. In this manuscript, 11 parameters of the HRV evaluation were analyzed. Therefore, the Bonferroni correction for a *p*-value was applied in the statistical analyses. According to Bonferroni correction, a *p*-value of < 0.0455 was considered statistically significant. The magnitude of the mean differences between the two groups was investigated using Cohen’s effect size [32]. Depending on the size of the Cohen’s d coefficient, the effect size was classified as small (d = 0.2), medium (d = 0.5), or large (d ≥ 0.8) [33]. The clinical characteristics of the RH and LH groups were compared using Mann–Whitney or chi2 tests when appropriate.

The statistical analysis was performed in the Python 3.8 environment using the scipy.stats package. The figures presented in this article and additional materials were created in the Python 3.8 environment using the seaborn package.

## 3. Results

The mean age among the studied cohort was similar, regardless of the affected hemisphere (RH: 64 ± 12 years vs. LH: 66 ± 13 years, *p* = 0.3). The other demographic data, index stroke characteristics, and vascular risk factors did not differ significantly between the groups with LH and RH stroke (Table 1). Additionally, the stroke severity assessed by NIHSS and functional disability assessed by mRS measures at baseline, discharge, and follow-up visits (30 days, 90 days, and 12 months following stroke onset) were similar between the LH and RH groups. The majority of the subjects (90% with LH stroke and 95% with RH stroke, *p* = 0.7) were functionally dependent during hospitalization and bedridden (mRS ≥ 4).

Before starting the analysis of heart rate variability, the percentage share of normal-to-normal intervals among all RR intervals was assessed. In total, 56 of the 64 subjects (87.5%) had more than 95% of the *NN* intervals in the entire record, 7 out of 64 subjects (11%) had between 90% and 95% of the intervals marked as *NN* among all RR intervals, and only 1 person out of 64 subjects (1.5%) had less than 90% of *NN* beats in the entire record (83.27% of *NN* intervals in 24 h of the RR recording; 16.73% ventricular beats). Although patients with atrial fibrillation and other cardiac arrhythmias were included in the study, the analysis of the percentage of normal-to-normal beats in the 24 h ECG record indicated that the majority of the group had less than 5% of the arrhythmias in the record. Finally, it was decided to compare the results of the HRV analysis performed at *NN* intervals (without taking into account the fragments of atrial fibrillation and other arrhythmias). Table 2 shows the numeric results in the X (Y) format, where X is the group mean, and Y is the standard deviation. The *p*-value columns represent Mann–Whitney statistical tests to compare the two groups (left hemisphere *NN* intervals vs. right hemisphere *NN* intervals). The effect size was determined using Cohen’s d method.

Additionally, a correction for multiple testing (Bonferroni correction) was applied. After applying the correction, the threshold value of the *p*-value was 0.05/11 = 0.00455. Table 2 shows (in bold) the measures that were found to be statistically significant after applying the Bonferroni correction.

The presented analyses were performed at different signal lengths for each patient. The purpose of this process was to obtain the longest possible excerpts from the records. Patients in the acute phase of stroke were characterized by significant values for the standard deviation of the meanNN (Table 2). As a result, cutting all signals to the same sample value would result in a significant signal loss for patients with a faster rhythm and lower mean NN. However, a test was carried out in which all signals were cut to the same number of samples while maintaining daytime and nighttime activities. The results confirmed the observations in Table 2. An analogous comparison of the values of the HRV analysis measurements for those signals with the same number of samples is included in the additional materials. Comparing the sample entropy values in both groups revealed statistically significant differences between the groups (*p*-value = 0.03, effect size = 0.7).

The Supplementary Materials include the box plot charts for all 11 parameters of the HRV analysis utilized in this work (Figures S2–S12). Figure 1 presents a box plot chart comparing sample entropy in the group of patients with left-sided and right-sided ischemic stroke, and the point plot shows the sample entropy values for all subjects.

Due to the influence of the circadian rhythm on the values of sample entropy [34], the daytime (6:00 a.m. to 10:00 p.m.) and nighttime (10:00 p.m. to 6:00 a.m.) fragments were separated from the records. The results are presented in Table 3.

Table 2 shows that the sample entropy determined based on the circadian recordings differentiating between left- and right-hemispheric stroke patients (*p*-value = 0.003). An analysis of the sample entropy marked on the daily HRV records also revealed the statistical significance (*p*-value = 0.017) of the results between the two hemispheres. The significance for the sample entropy of the HRV fragments overnight was (*p*-value = 0.076). However, these results were only slightly above the threshold of statistical significance (*p*-value = 0.05).

The Supplementary Materials include a graph (Figure S1) showing the Spearman correlation between the individual parameters of the HRV analysis (in the time domain, in the frequency domain, and nonlinear) and the hemispheric location of the stroke. The highest correlation was observed for the sample entropy values (correlation coefficient = 0.38, *p*-value = 0.002). Moreover, the pNN50 (correlation coefficient = 0.29, *p*-value = 0.018) and RMSSD (correlation coefficient = 0.24, *p*-value = 0.06) results correlated well with the location of the stroke.

## 4. Discussion

It is commonly believed that HRV analysis is a good method for describing the activity of the autonomic nervous system. Currently, the analysis of heart rate variability may be of diagnostic value not only in the case of patients in the acute phase of stroke, but also in patients treated with reperfusion in the acute phase of myocardial infarction [35]. The scientific literature on this subject indicates correlations between HRV and stroke mortality [10,11], recovery, and short- or long-term complications [9]. Although the exact mechanisms have not yet been fully established, several studies suggested anatomical asymmetry between the right and left cerebral hemispheres in the modulation of autonomic nervous system activity in the central nervous system [36].

Our pilot study provides more data on the important differences in HRV measures between patients with severe RH and LH stroke. Importantly, our cohort with LH and RH stroke was well-balanced, showing a similar distribution of stroke risk factors and baseline stroke severity when assessed using functional and radiological scales (mRS, NIHSS, and ASPECTS), which distinguishes the present research from previous studies. Although the classical methods of linear HRV analysis (in both the time and frequency domains) did not reveal any significant statistical differences between RH and LH stroke, the nonlinear analysis of sample entropy showed significant statistical differences between these groups (*p*-value = 0.003, Bonferroni corrected *p*-value = 0.033, effect size = 0.8). Interestingly, patients with RH involvement were characterized by a lower mean value for sample entropy [RH: 0.92 (0.46) vs. LH: 1.31(0.53)], which may suggest a lower complexity of HRV in this group compared to the LH stroke patients. The clinical significance of this finding is not clear. However, decreased HRV complexity was also observed in patients with type 1 diabetes mellitus or congestive heart failure, indicating that this phenomenon may reflect underlying systemic hemodynamic turbulences.

The Mann–Whitney statistical significance test revealed that sample entropy and pNN50 were indicators that differentiated LH and RH stroke localization. However, the Bonferroni correction excluded the statistical significance of the pNN50 parameter.

In a previous article by Klingelhöfer and Sander [37], the authors observed lateralization in the activation of the sympathetic nervous system, according to the brain lesion side. Subjects with right hemispheric stroke experienced increased sympathetic activity, while those with left-hemisphere stroke had increased parasympathetic activity.

An analysis of the sample entropy results presented in the current paper (Table 2) demonstrated that subjects with a right-hemisphere stroke experienced lower sample entropy values, i.e., they had lower HRV complexity. Similar observations were demonstrated by Naver et al. [20], who found that RH stroke subjects experienced reduced respiratory HRV compared to the LH and control groups. Porta et al. [38] published a study searching for a physiological interpretation of sample entropy. The authors used a graded head-up tilt test to observe the behavior of the sympathetic and parasympathetic systems and the values of sample entropy. The results of their study indicated that the lower the complexity (lower entropy value), the higher the activity of the sympathetic nervous system. Additionally, patients with a stroke in the right hemisphere had higher LF_nu_ values and LF/HF ratios, while subjects suffering from a stroke in the left hemisphere had higher RMSSD, pNN50, and HF_nu_ values. Referring to the physiological interpretation of linear analysis measures in the time domain [39], it can be concluded that higher RMSSD and pNN50 values indicate higher activity in the parasympathetic system. The pNN50 measure is strongly correlated with RMSSD and HF [39]. Observations of RMSSD determined via 24 h HRV records indicate correlations with pNN50 and HF. The RMSSD, however, is more strongly influenced than the SDNN by the parasympathetic system. The SDNN measure is related to both sympathetic and parasympathetic activities [39]. It is commonly believed that the high-frequency spectral power component describes the activity of the parasympathetic system and that LF is related to the sympathetic system [15]. However, such an assignment raises many objections [40,41]. In [42], Billman refuted the hypothesis that the LF/HF ratio can quantify “sympatho-vagal balance”, noting that it is impossible to determine with complete certainty the physiological interpretation of LF/HF. Porta et al. [38] indicated that the power of short-term heart period variability in normalized HF and LF correlates with the sympathetic nervous system.

The correlation matrix between the HRV analysis parameters included in the additional materials indicates that sample entropy correlates with RMSSD (correlation coefficient = 0.45, *p*-value < 0.001), pNN50 (correlation coefficient = 0.53, *p*-value < 0.001), and HF_nu_ (correlation coefficient = 0.47, *p*-value < 0.001) but has anti-correlation with LF_nu_ (correlation coefficient = −0.47, *p*-value < 0.001) and LF/HR (correlation coefficient = −0.47, *p*-value < 0.001). The correlation between sample entropy and the RMSSD, pNN50, and HF_nu_ parameters and the anti-correlation with LF_nu_ and LF/HF may represent further evidence supporting the hypothesis that higher sample entropy corresponds to higher activity in the parasympathetic system.

In the present study, we conducted the main analyses on 24 h records without division into day and night periods because the studied group consisted of hospitalized patients in the acute phase of stroke, who usually do not have clearly marked day and night activities. The majority of subjects were bedridden and could not have their sleeping times reliably recorded due to their functional disabilities (e.g., aphasia). An additional analysis of the sample entropy values by day and night time (Table 3) showed that the daily records also statistically significantly differentiated between patients with right- and left-hemispheric stroke (*p*-value = 0.017). Sample entropy determined on the nighttime HRV recordings slightly exceeded the adopted threshold of statistical significance (*p*-value = 0.076).

A question remains: why does sample entropy act to differentiate these patients so well? To answer this question, we assessed whether the sample entropy parameter would still be valid after the removal of persons with atrial fibrillation/modification of the ‘*m*’ and ‘*r*’ parameters, i.e., pattern length and the tolerance/analysis of shorter time series (day or night only). After this adjustment, the sample entropy parameter still correctly differentiated patients with right-hemisphere stroke from those with left-hemisphere stroke. The observations showed that sample entropy in each of the above-mentioned cases differentiated those patients with left from those with right-hemisphere stroke. Additionally, right-hemisphere stroke survivors had lower HRV complexity (lower sample entropy) than left-hemisphere stroke survivors.

However, our study has some limitations. First, the results are based on a retrospective and small sample. Second, the study population included patients with acute and severe ischemic stroke, thus limiting the generalizability of our data to other stroke populations, including hemorrhagic, minor, or chronic stroke patients. Third, our study did not include a control group, so we could not determine the effect of a stroke on HRV variability compared to healthy subjects.

## 5. Conclusions

Heart rate variability analyses can provide important information to help identify the hemispheric location of an ischemic stroke. Right hemispheric stroke patients were characterized by lower HRV complexity (lower sample entropy) compared to the left-sided stroke group. Further studies assessing this phenomenon, which include larger cohorts with different stroke etiologies, are, therefore, warranted.

## Figures and Tables

**Figure 1:**
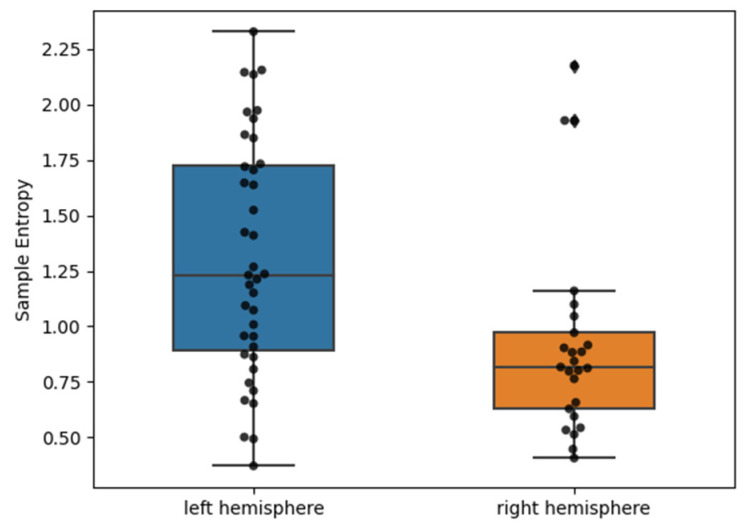
Boxplot comparing sample entropy values in the group of subjects with left-hemispheric ischemic stroke vs. those with right-hemispheric ischemic stroke. Circle points mark the values of the sample entropy individuals and a diamond sign defines outliers.

**Table 1:** Baseline characteristics of the studied group.

	Left-Hemisphere Stroke	Right-Hemisphere Stroke	*p*-Value *
*n*	39 (61%)	25 (39%)	
Mean Age (SD)	66 (13)	64 (12)	0.3
Sex (f) *n* (%)	20 (51%)	12 (48%)	0.8
AF (%)	15 (37%)	5 (20%)	0.1
Hypertension (%)	25 (64%)	17 (68%)	0.7
Diabetes (%)	7 (18%)	3 (12%)	0.67
Smoking (%)	14 (36%)	9 (36%)	0.99
CHD (%)	6 (15%)	4 (16%)	0.9
HF (%)	9 (23%)	3 (12%)	0.26
Dyslipidemia (%)	7 (18%)	4 (16%)	0.84
Past Stroke or TIA (%)	6 (15%)	3 (12%)	0.9
ASPECTS (SD)	7.77 (2.03)	7.52 (2.59)	0.3
sICH (%)	3 (8%)	5 (20%)	0.6
NIHSS baseline (SD)	15.49 (5.79)	14.46 (4.15)	0.2
NIHSS discharge (SD)	7.89 (5.85)	5.83 (6.09)	0.13
In-hospital mortality (%)	2 (5%)	1 (4%)	0.83
mRS discharge (SD)	3.32 (1.73)	2.88 (1.61)	0.2
mRS 30 days (SD)	3.21 (1.66)	2.84 (1.59)	0.19
mRS 90 days (SD)	3 (1.90)	2.72 (1.76)	0.4
mRS 12 months (SD)	2.94 (1.98)	2.33 (1.97)	0.2
Antiarrhythmic Drugs			
Digoxin (%)	3 (8%)	1 (4%)	0.55
Beta blocker (%)	24 (62%)	14 (56%)	0.67
Other (%)	2 (6%)	1 (4%)	0.7

Values are the mean ± SD for quantitative variables and n (%) for qualitative variables. Abbreviations: AF—atrial fibrillation; ASPECTS—Alberta stroke program early CT score; CHD—coronary heart disease; HF—heart failure; sICH—secondary intracranial hemorrhage; TIA—transient ischemic attack; mRS—Modified Rankin Scale; NIHSS—The National Institutes of Health Stroke Scale. * Mann–Whitney or chi2 tests, where applicable.

**Table 2:** Comparison of linear and nonlinear parameters of the HRV analysis between the left hemisphere (LH) and right hemisphere (RH) ischemic stroke groups.

Parameter	Left-Hemisphere Stroke NN Intervals (*n =* 39)	Right-Hemisphere Stroke NN Intervals (*n =* 25)	*p*-Value (LH_NN_ vs. RH_NN_)	Effect Size (Cohen’s d)
Time-based analysis				
MeanNN [ms]	843 (123)	854 (174)	0.879	−0.1
SDNN [ms]	137 (60)	113 (38)	0.117	0.4
RMSSD [ms]	113 (81)	76 (61)	0.061	0.5
pNN50 [%]	33.35 (28.54)	18.52 (23.75)	0.020	0.5
Frequency-based analysis				
LH/HF	1.68 (2.50)	2.24 (2.87)	0.110	−0.2
HFnu [%]	48.42 (16.41)	42.66 (17.88)	0.110	0.3
LFnu [%]	51.58 (16.41)	57.34 (17.88)	0.110	-0.3
Nonlinear analysis				
GI [%]	52.31 (2.42)	53.28 (3.43)	0.289	−0.3
PI [%]	50.70 (1.86)	51.37 (3.31)	0.536	−0.3
**Sample entropy**	**1.31 (0.53)**	**0.92 (0.46)**	**0.003**	**0.8**
α_1_	0.90 (0.25)	1.00 (0.27)	0.187	−0.4

**Table 3:** Sample entropy values during the day and at night among the group of patients with left-hemisphere (LH) and right-hemisphere (RH) ischemic stroke.

	Sample Entropy (LH))	Sample Entropy (RH)	*p*-Value (LH_NN_ vs. RH_NN_)	Effect Size (Cohen’s d)
Day	1.26 (0.54)	0.94 (0.53)	0.017	0.6
Night	1.40 (0.48)	1.17 (0.53)	0.076	0.5

## Data Availability

The datasets used and/or analyzed during the current study are available from the corresponding author on reasonable request.

## Supplementary Materials

The following supporting information can be downloaded at: https://www.mdpi.com/article/10.3390/life12101659/s1. Figure S1: The matrix of mutual correlations of the parameters of HRV analysis (in the time, frequency, and nonlinear domains), and the correlation of the location of the stroke with the parameters of the HRV analysis.; Figure S2: Boxplot comparing meanNN values in the group of subjects with LF vs. RH.; Figure S3: Boxplot comparing SDNN values in the group of subjects with LF vs. RH.; Figure S4: Boxplot comparing RMSSD values in the group of subjects with LF vs. RH.; Figure S5: Boxplot comparing pNN50 values in the group of subjects with LF vs. RH.; Figure S6: Boxplot comparing HF_nu_ values in the group of subjects with LF vs. RH.; Figure S7: Boxplot comparing LF_nu_ values in the group of subjects with LF vs. RH.; Figure S8: Boxplot comparing GI (Guzik index) values in the group of subjects with LF vs. RH.; Figure S9: Boxplot comparing PI (Porta index) values in the group of subjects with LF vs. RH.; Figure S10: Boxplot comparing Sample Entropy values in the group of subjects with LF vs. RH.; Figure S11: Boxplot comparing α_1_ values in the group of subjects with LF vs. RH.; Figure S12: Boxplot comparing α_2_ values in the group of subjects with LF vs. RH. In Figures S2–S12 the circle points mark the values of the sample entropy individuals and a diamond sign defines outliers.
