# Supplementary figures and images for: The Hemisphere of the Brain in Which a Stroke Has Occurred Visible in the Heart Rate Variability
Source: Life (Basel). 2022 Oct 20;12(10):1659. doi: 10.3390/life12101659 (PMC9605232; doi:10.3390/life12101659)

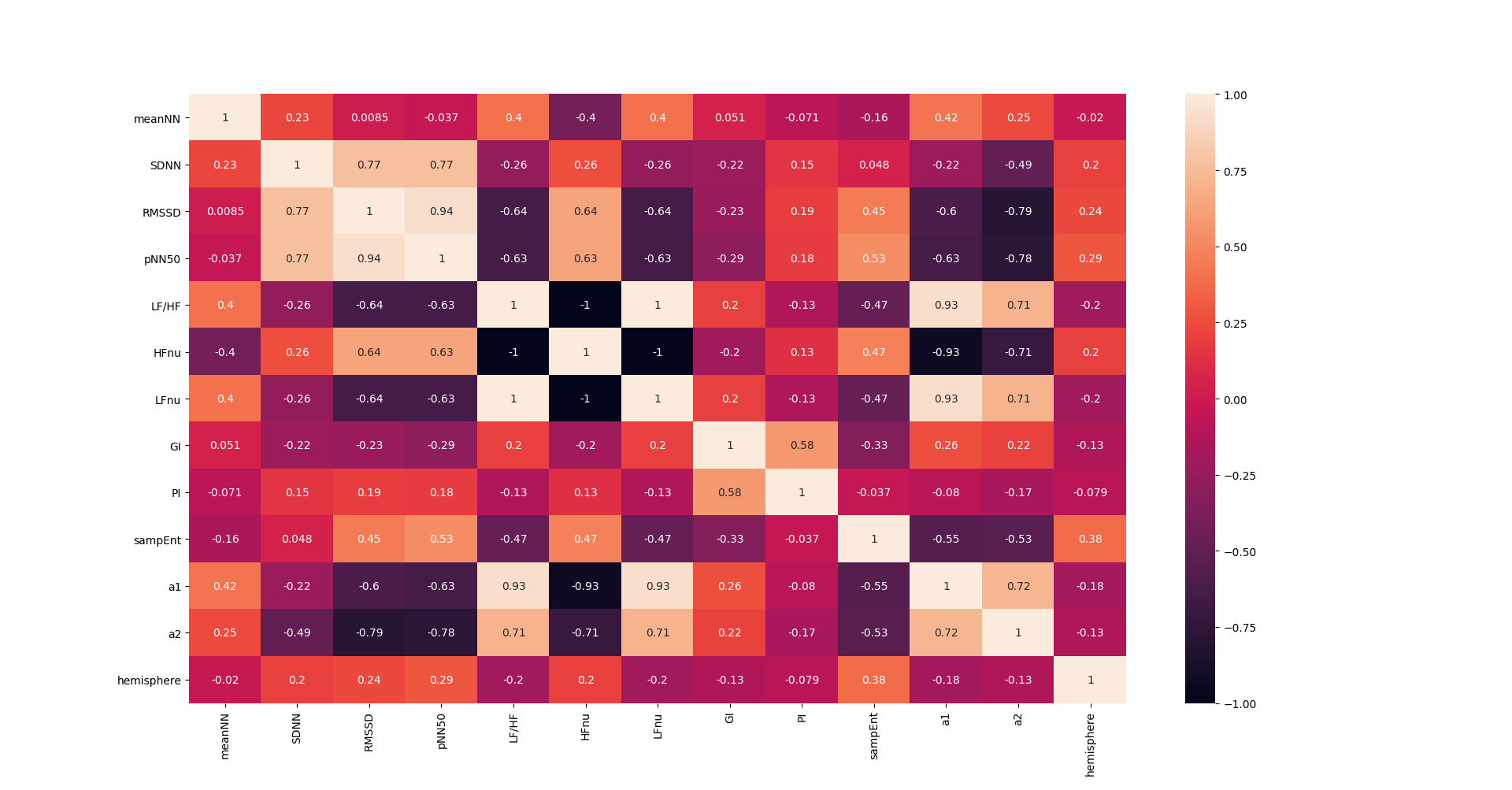

Supplement: Supplementary file 1 [file life-12-01659-s001.zip › Supplementary_Materials/Figure S1: Correlations.png]

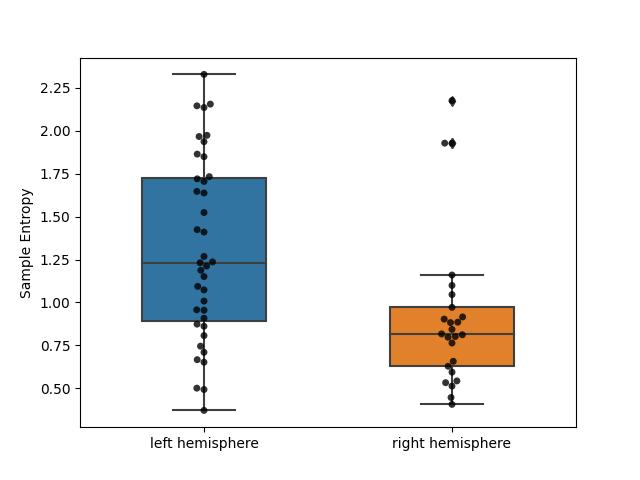

Supplement: Supplementary file 1 [file life-12-01659-s001.zip › Supplementary_Materials/Figure S10: Box plot_SampleEntropy.jpg]

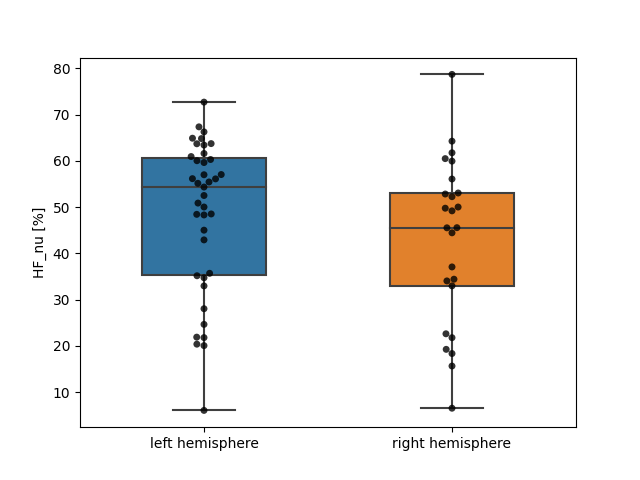

Supplement: Supplementary file 1 [file life-12-01659-s001.zip › Supplementary_Materials/Figure S6: Box plot_HF_nu.png]

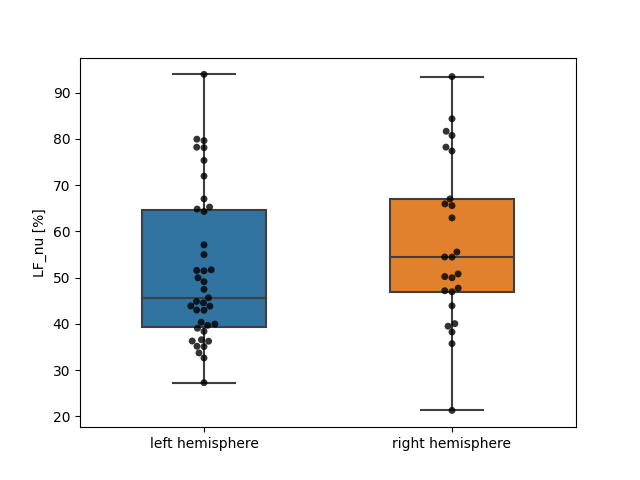

Supplement: Supplementary file 1 [file life-12-01659-s001.zip › Supplementary_Materials/Figure S7: Box plot_LF_nu.png]

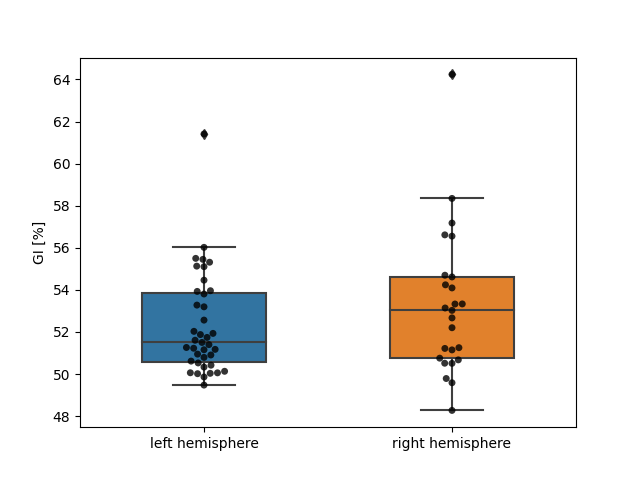

Supplement: Supplementary file 1 [file life-12-01659-s001.zip › Supplementary_Materials/Figure S8: Box plot_GI.png]

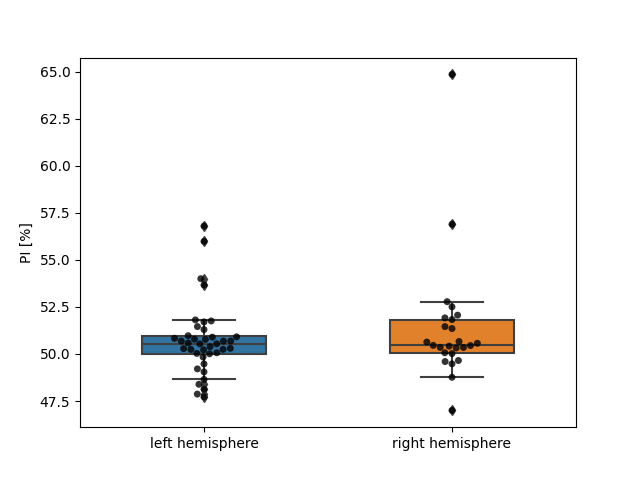

Supplement: Supplementary file 1 [file life-12-01659-s001.zip › Supplementary_Materials/Figure S9: Box plot_PI.png]

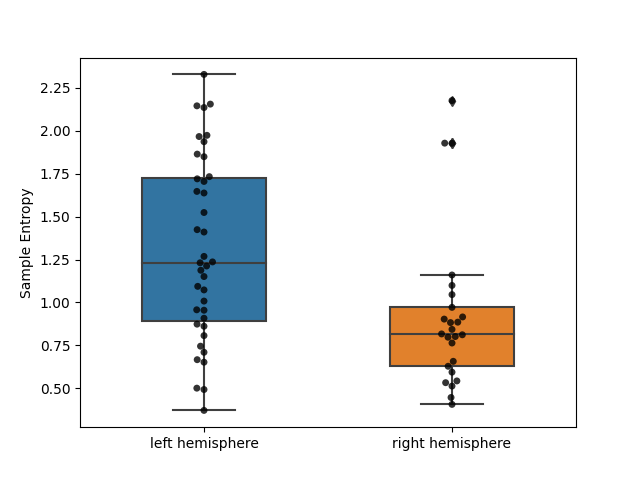

Supplement: Supplementary file 1 [file life-12-01659-s001.zip › Supplementary_Materials/Figure S10: Box plot_SampleEntropy.png]

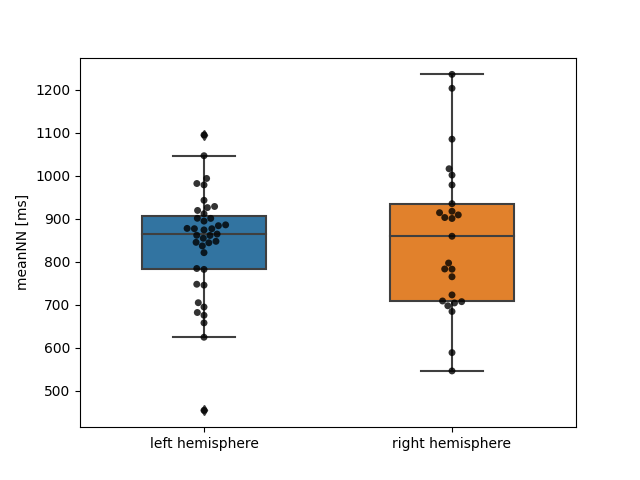

Supplement: Supplementary file 1 [file life-12-01659-s001.zip › Supplementary_Materials/Figure S2: Box plot_meanNN.png]

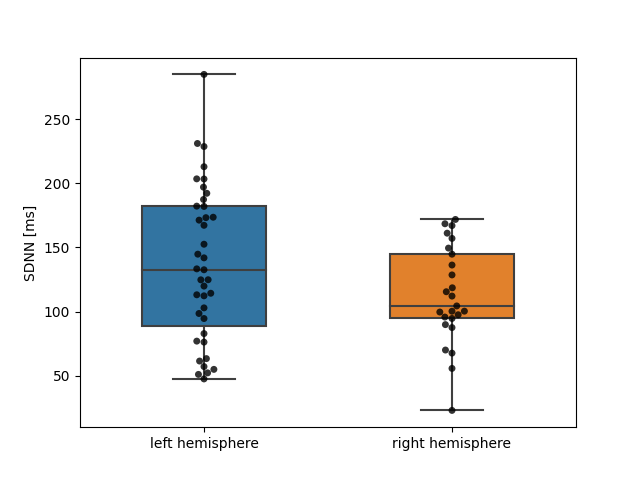

Supplement: Supplementary file 1 [file life-12-01659-s001.zip › Supplementary_Materials/Figure S3: Box plot_SDNN.png.png]

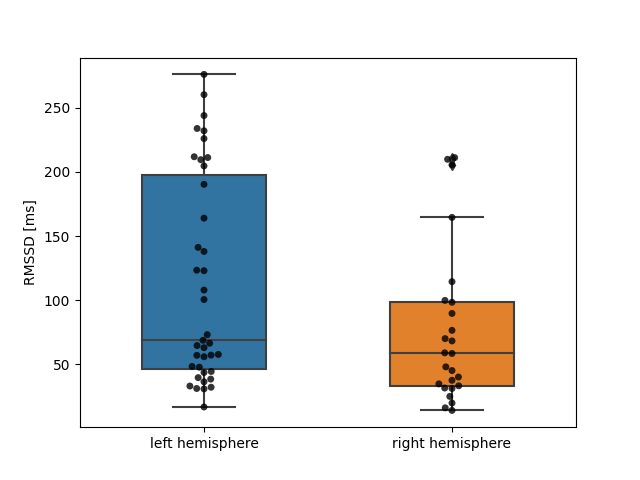

Supplement: Supplementary file 1 [file life-12-01659-s001.zip › Supplementary_Materials/Figure S4: Box plot_RMSSD.png]

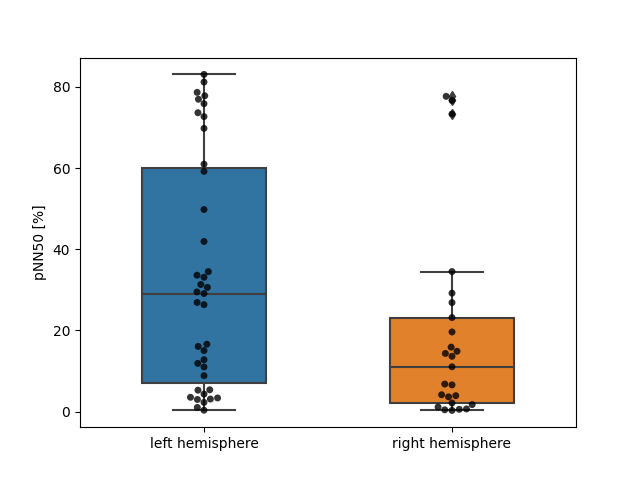

Supplement: Supplementary file 1 [file life-12-01659-s001.zip › Supplementary_Materials/Figure S5: Box plot_pNN50.png]

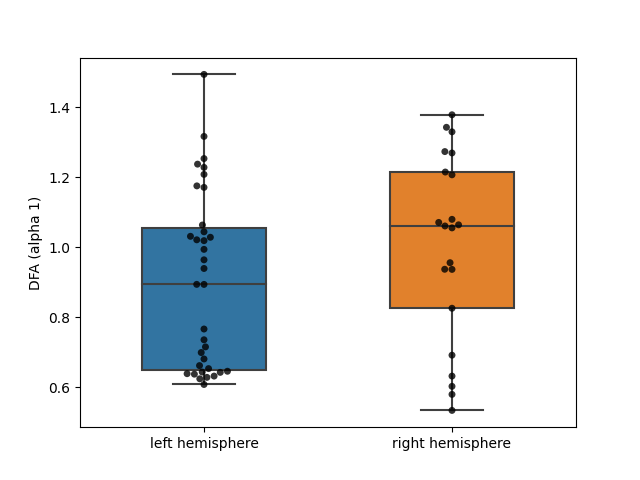

Supplement: Supplementary file 1 [file life-12-01659-s001.zip › Supplementary_Materials/Figure S11: Box plot_DFA_alpha1.png]

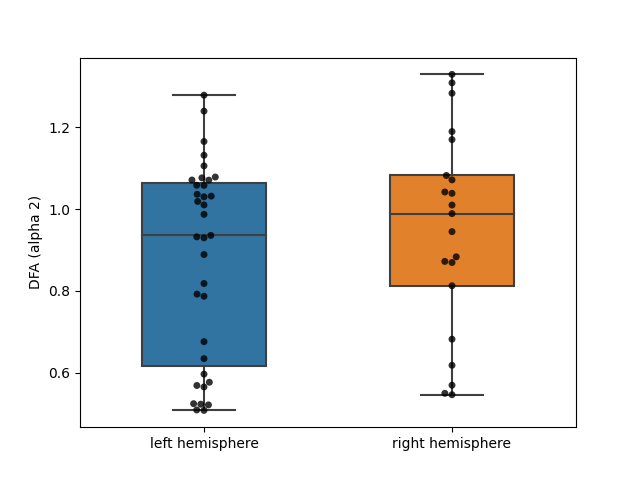

Supplement: Supplementary file 1 [file life-12-01659-s001.zip › Supplementary_Materials/Figure S12: Box plot_DFA_alpha2.png]
